# Design and Experimental Field Mapping of Merritt Coil System for Polarized ^3^He Precision Measurements

**DOI:** 10.3390/s26154898

**Published:** 2026-08-03

**Authors:** Ruoyun Wen, Chaoyang Zhao, Haiyang Yan

**Affiliations:** 1 College of Physics, Sichuan University, Chengdu 610065, China; wenruoyun@stu.scu.edu.cn; 2 Key Laboratory of Radiation Physics and Technology of the Ministry of Education, Institute of Nuclear Science and Technology, Sichuan University, Chengdu 610064, China; 3 Institute of Fundamental Physics and Quantum Technology, and School of Physical Science and Technology, Ningbo University, Ningbo 315211, China

**Keywords:** Merritt coils, polarized helium-3, magnetic-field uniformity, noble gas magnetometer, spin relaxation, precision measurement, magnetic field mapping

## Abstract

Highly polarized ^3^He is widely used in precision magnetometry, neutron spin filtering, and fundamental symmetry tests, where magnetic field gradients can shorten relaxation and coherence times and introduce systematic uncertainties. We present the design, modeling, and experimental characterization of a compact four-square-coil Merritt system developed to provide a uniform holding field for precision measurements of polarized ^3^He. The magnetic field is calculated directly from analytical vector potentials and analyzed using a near-center expansion. By combining Maxwell’s equations with the spatial symmetries of square- and circular-coil systems, we show that the dominant second-order transverse gradients within the small central cell volume are determined by the axial curvature of $B_{z}$. The relevant gradient performance can therefore be characterized using a single axial scan, without routine complete three-dimensional mapping. The calculated performance of the Merritt configuration is quantitatively compared with that of the Helmholtz, Lee–Whiting, and Garrett systems. A prototype is constructed and measured using a three-axis fluxgate magnetometer mounted on a motorized translation stage.The measured axial field agrees well with the calculated profile, and the normalized magnetic field gradient in the central 10 cm region is below $10^{-4}$ cm^−1^. This region fully covers the cylindrical ^3^He cell, whose diameter and length are both no greater than 5 cm. The Merritt configuration therefore provides a practical compromise between gradient suppression, compactness, optical access, and mechanical simplicity for polarized noble- gas sensors and precision measurements.

## 1. Introduction

Polarized noble gases provide long-lived nuclear spin ensembles for precision magnetometry, inertial sensing, neutron spin filtering, and tests of fundamental symmetries and exotic spin-dependent interactions [1,2,3,4]. Among them, polarized ^3^He is especially attractive because of its spin-1/2 nuclear structure, long longitudinal relaxation time [5,6], and compatibility with spin exchange and metastability exchange optical pumping methods [1,7,8].

Two characteristic time scales are particularly important in polarized ^3^He precision measurements. The longitudinal relaxation time $T_{1}$ characterizes the loss of nuclear spin polarization along the holding-field direction, whereas the transverse relaxation time $T_{2}$ characterizes the decay of transverse magnetization and phase coherence during spin precession. A long $T_{1}$ is required to preserve the nuclear polarization throughout the measurement, while a long $T_{2}$ provides a narrow spin precession linewidth and improved frequency sensitivity. Both quantities may be limited by wall relaxation, dipole–dipole interactions, and magnetic field inhomogeneity [9,10,11,12]. In particular, as ^3^He atoms diffuse through an inhomogeneous magnetic field, they experience time-dependent transverse-field components that contribute to longitudinal relaxation, transverse decoherence, and line broadening.

For a polarized ^3^He cell in a nominally uniform holding field along $\hat{z}$, small transverse magnetic-field gradients contribute to longitudinal relaxation. As a simple example, the magnetic field may be written as

$$B=B_{0}\hat{z}+gx\hat{x}+gy\hat{y}-2gz\hat{z},$$

where *g* characterizes the small residual field gradient. This field satisfies both ∇·B=0 and ∇×B=0 in the source-free central region. Within Redfield theory, the longitudinal relaxation rate can be expressed as [4,11]

(1) $$\frac{1}{T_{1}}=\frac{\gamma^{2}}{2}\left[S_{B_{x}}\left(\omega_{0}\right)+S_{B_{y}}\left(\omega_{0}\right)\right],$$

where

$$S_{B_{x}}\left(\omega \right)=\int_{-\infty }^{\infty }\langle B_{x}\left(t\right)B_{x}\left(t+\tau \right)\rangle e^{-i\omega \tau }\;d\tau ,$$

and

$$S_{B_{y}}\left(\omega \right)=\int_{-\infty }^{\infty }\langle B_{y}\left(t\right)B_{y}\left(t+\tau \right)\rangle e^{-i\omega \tau }\;d\tau .$$

Here, γ is the gyromagnetic ratio of the ^3^He nucleus, and $\omega_{0}=\gamma B_{0}$ is the Larmor frequency. The correlation functions describe the magnetic field fluctuations experienced by the nuclear spins as the atoms undergo random diffusive motion inside the cell.

For the simple linear-gradient model given above, $B_{x}=gx$, and $B_{y}=gy$. Equation (1) then becomes

(2) $$\frac{1}{T_{1}}=\frac{\gamma^{2}}{2}g^{2}\left[S_{x}\left(\omega_{0}\right)+S_{y}\left(\omega_{0}\right)\right],$$

where

$$S_{x}\left(\omega \right)=\int_{-\infty }^{\infty }\langle x(t)x(t+\tau )\rangle e^{-i\omega \tau }\;d\tau ,$$

and

$$S_{y}\left(\omega \right)=\int_{-\infty }^{\infty }\langle y(t)y(t+\tau )\rangle e^{-i\omega \tau }\;d\tau .$$

The position autocorrelation functions, such as $\langle x(t)x(t+\tau )\rangle$, are determined by the diffusion of ^3^He atoms in the cell. They depend on the cell geometry, gas pressure, and temperature, which are usually fixed once the cell has been fabricated and filled. Therefore, for a given ^3^He cell and operating condition, reducing the transverse magnetic-field gradient *g* is an effective way to suppress the corresponding contribution to $1/T_{1}$. Equation (2) thus motivates optimizing the coil geometry to minimize transverse gradients within the central cell volume.

Helmholtz coils are widely used to generate nearly uniform magnetic fields. Their principal advantages are structural simplicity and straightforward alignment. However, although the second-order axial variation is canceled, the remaining fourth-order terms can produce appreciable gradients away from the center. Higher-order circular configurations, such as the Lee–Whiting and Garrett systems, provide stronger suppression of leading-field variations [13,14]. This improved field performance is accompanied by increased mechanical complexity: these systems require multiple coils with unequal axial positions and turn numbers, and the Garrett configuration additionally requires coils with different radii.

Square-coil systems, especially Merritt-type configurations [15], provide a practical alternative because they are easier to fabricate, align, and integrate with optical pumping and detection systems. Merritt and related four-square-coil systems have also been used in biological exposure and biomagnetic experiments [16,17]. These studies illustrate the broader applicability of square-coil systems. The present application, however, has a different primary requirement: rather than maximizing the size of a conventionally uniform exposure region, it seeks to suppress the normalized magnetic-field gradients that govern spin relaxation and coherence within the comparatively small volume occupied by the polarized ^3^He cell.

The present work focuses on the specific requirements of polarized noble gas experiments, which differ from those of many other uniform-field applications. In a typical polarized ^3^He apparatus, the cell dimension is approximately one-tenth of the characteristic coil size, and the principal magnetic field requirement is to suppress the relative gradients that drive nuclear spin relaxation. At the same time, the coil system must preserve access for optical pumping and probe beams, provide space for the ^3^He cell, oven, and RF coils, remain compatible with magnetic shielding, maintain a low heat load, and be straightforward to fabricate and align.

The aim of this work is to establish and experimentally validate a compact Merritt coil system and an axial-scan-based method for determining the dominant near-center magnetic-field gradients relevant to polarized ^3^He precision measurements, while assessing its performance relative to the Helmholtz, Lee–Whiting, and Garrett configurations.

## 2. Field Uniformity Requirement and Coil Field Model

### 2.1. Scaling Property of Circular and Square Current Loops

The magnetic field of a current loop has a useful scale property. For a circular loop of radius *a*, the vector potential can be expressed in terms of the dimensionless coordinate $\bar{r}=r/a$. After scale transformation, the magnetic field at a geometrically corresponding point scales as 1/a. Thus, the field distribution needs to be calculated only once for a unit radius loop, and other sizes are obtained by simple scaling.

An analogous result holds for a square loop. For a square loop with side length 2a, define normalized coordinates

(3) $$\bar{x}=x/a,\;\bar{y}=y/a,\;\bar{z}=z/a.$$

**Figure 1:**
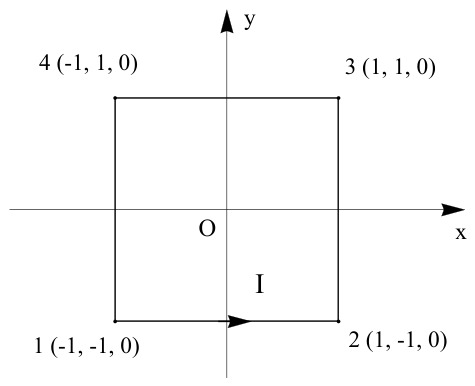
Definition of the vertex order and coordinate system for a square coil. Here, *I* denotes the loop current.

The vector potential of the square loop has only *x* and *y* components. As shown in Figure 1, for the normalized square loop with vertices at (±1,±1,0), define

(4) $$\begin{array}{c} r_{1}=\sqrt{{\left(1+x\right)}^{2}+{\left(1+y\right)}^{2}+z^{2}},\;\;\;r_{2}=\sqrt{{\left(1-x\right)}^{2}+{\left(1+y\right)}^{2}+z^{2}},\\ r_{3}=\sqrt{{\left(1-x\right)}^{2}+{\left(1-y\right)}^{2}+z^{2}},\;\;\;r_{4}=\sqrt{{\left(1+x\right)}^{2}+{\left(1-y\right)}^{2}+z^{2}}. \end{array}$$

The vector potential can be written as

(5) $$\begin{array}{cc} A_{x} & =\frac{\mu_{0}I}{4\pi }\ln\frac{\left(r_{2}+1-x\right)\left(r_{4}-1-x\right)}{\left(r_{1}-1-x\right)\left(r_{3}+1-x\right)}, \end{array}$$

(6) $$\begin{array}{cc} A_{y} & =\frac{\mu_{0}I}{4\pi }\ln\frac{\left(r_{3}+1-y\right)\left(r_{1}-1-y\right)}{\left(r_{2}-1-y\right)\left(r_{4}+1-y\right)}. \end{array}$$

The magnetic field is then obtained from

(7) $$B_{x}=-\frac{\partial A_{y}}{\partial z},\;B_{y}=\frac{\partial A_{x}}{\partial z},\;B_{z}=\frac{\partial A_{y}}{\partial x}-\frac{\partial A_{x}}{\partial y}.$$

For an arbitrary square half-side length *a*, the normalized field pattern is preserved, and the field amplitude scales as 1/a for a fixed current.

General-purpose finite-element packages can also be used to calculate the field of this coil system. However, the present experiment requires resolving relative field variations at the $10^{-5}$ level: for a field of approximately 5 G, the nominal 10 nT resolution of the fluxgate magnetometer corresponds to a relative scale of approximately $2\times 10^{-5}$. A preliminary finite-element calculation available to us resolved the relative field variation only at approximately the $10^{-2}$ level because it had been configured for substantially less stringent engineering requirements. This limitation reflects the numerical settings and intended accuracy of that particular calculation rather than an intrinsic limitation of finite-element software.

For the geometrically simple current loops considered here, we therefore calculate the field directly from analytical magnetic vector potentials. The vector potential of a circular current loop is well-known [18], while the corresponding expressions for a square loop are given explicitly in Equations (5) and (6). For a winding represented by *N* individual loops, the total vector potential is obtained by superposition, $A_{\mathrm{tot}}\left(r\right)=\sum_{n=1}^{N}A_{n}\left(r\right)$, and the magnetic field follows from $B=\nabla \times A_{\mathrm{tot}}$. In the numerical evaluation, the actual position and size of each turn are included. This direct loop-by-loop calculation is transparent and computationally efficient for the present geometry and avoids the need to select a finite-element mesh, artificial computational boundaries, and convergence criteria.

### 2.2. Near-Center Expansion for Symmetric Square-Coil Systems

For a system of square current loops sharing a common symmetry axis, the vector potential near the center is constrained by mirror symmetries. To the fourth order in the normalized coordinates, the field can be written in the form

(8) $$B_{x}=-2b_{00}xz-2b_{02}xy^{2}z-2b_{20}x^{3}z-4c_{00}xz^{3},$$

(9) $$B_{y}=-2b_{00}yz-2b_{02}x^{2}yz-2b_{20}y^{3}z-4c_{00}yz^{3},$$

(10) $$B_{z}=2a_{00}-b_{00}\left(x^{2}+y^{2}\right)-\frac{1}{2}b_{20}\left(x^{4}+y^{4}\right)+b_{02}x^{2}y^{2}+2b_{00}z^{2}-6c_{00}\left(x^{2}+y^{2}\right)z^{2}+2c_{00}z^{4}.$$

These expressions already include the constraints imposed by ∇·B=0 and ∇×B=0 in the source-free central region. In the small central volume used for the ^3^He cell, the second-order terms dominate, giving

(11) $$B_{x}≃-2b_{00}xz,\;B_{y}≃-2b_{00}yz,\;B_{z}≃B_{0}-b_{00}\left(x^{2}+y^{2}\right)+2b_{00}z^{2}.$$

Thus, to leading order, the transverse field components and the corresponding transverse gradients relevant to Equation (11) are determined by the axial curvature of $B_{z}$.

The same second-order structure is also obtained for circular coils. In other words, the near-center magnetic-field expansion of a symmetric square-coil system has the same form as that of an axially symmetric circular-coil system to the lowest nontrivial order. This equivalence follows from the spatial symmetries of the coil system together with Maxwell’s equations in the current-free region. Up to the second order, the field can be written as

(12) $$B_{x}=-cxz,\;B_{y}=-cyz,\;B_{z}=B_{0}+cz^{2}-\frac{c}{2}\left(x^{2}+y^{2}\right),$$

where the coefficient *c* is determined by the axial variation in $B_{z}$.

However, this equivalence no longer holds at higher orders. A circular-coil system has continuous axial symmetry, so the magnetic field near the axis is fully determined by the axial field $B_{z}\left(0,0,z\right)$. In contrast, a square-coil system has only fourfold rotational symmetry. As a result, fourth-order anisotropic terms such as $x^{4}+y^{4}$, $x^{2}y^{2}$, and $\left(x^{2}+y^{2}\right)z^{2}$ are allowed. Therefore, the magnetic field of a square-coil system cannot, in general, be completely determined from the axial field alone beyond the lowest-order expansion.

This observation provides a practical field characterization method for polarized ^3^He experiments. Although the complete higher-order field of a square-coil system cannot be reconstructed from the axial field alone, the ^3^He cell occupies only a small central volume, with a characteristic dimension of approximately one-tenth of the coil size. In this region and provided that the relevant expansion coefficients are sufficiently small, the second-order terms dominate, and the field is accurately described by Equation (11). The dominant transverse field components and gradients are therefore determined by the same coefficient that governs the axial curvature of $B_{z}$.

This observation provides a practical field characterization method for polarized ^3^He experiments. Although the complete higher-order field of a square-coil system cannot be reconstructed from the axial field alone, the ^3^He cell occupies only a small central volume, with a characteristic dimension of approximately one tenth of the coil size. The spatial dependence of the fourth-order terms is therefore suppressed by an additional factor of approximately ${\left(0.1\right)}^{2}$ compared with that of the second-order terms.In this region, and provided that the relevant expansion coefficients are not anomalously large, the second-order terms dominate, and the field is accurately described by Equation (11). The dominant transverse field components and gradients are therefore determined by the same coefficient that governs the axial curvature of $B_{z}$.

### 2.3. Comparison of Candidate Coil Configurations

**Figure 2:**
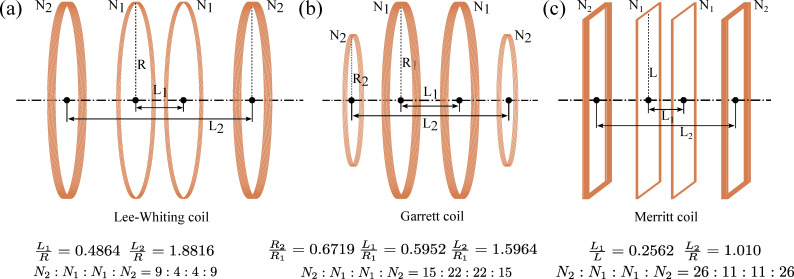
The Parameters for three types of coil configurations.

Several standard coil configurations were considered for producing a highly uniform holding field for polarized ^3^He experiments. A Helmholtz pair cancels the second-order axial field variation, thereby providing fourth-order axial uniformity. However, its higher-order coefficients are relatively large, and the transverse gradients can become significant away from the central point. Four Garrett-type circular coils can further suppress the low-order gradients and provide excellent field uniformity, but they generally require coils of different radii and nontrivial turn ratios, which complicate fabrication and alignment. The Lee–Whiting four-coil system (Figure 2a) uses equal-radius circular coils and can increase the useful volume for certain uniformity thresholds, but it does not minimize the transverse gradient term that is most relevant to Equation (2). The Merritt four-square-coil system offers a favorable compromise between field uniformity and mechanical simplicity and is therefore well-suited for the present experimental requirements.

A quantitative comparison of the four candidate configurations is summarized in Table 1. To provide a consistent estimate, the normalized axial gradient is evaluated from the leading axial field term at $|\bar{z}|=0.1$, where $\bar{z}$ denotes the axial coordinate normalized to the characteristic coil size.

For comparison, the Helmholtz coil expansion near the center is

$$B_{z}=1-1.152z^{4}+3.456z^{2}\left(x^{2}+y^{2}\right)+\ldots .$$

Although the second-order terms are completely canceled, the fourth-order coefficients are at the level of ∼1. As a result, for points located at about 1/10 of the coil size away from the center, the relative gradient can reach the $10^{-3}$ level. Therefore, the Helmholtz configuration is not optimal for the long-relaxation-time polarized ^3^He experiments considered here.

In the Lee–Whiting configuration (Figure 2a), four circular current loops of radius 1 are arranged at the axial positions ±0.2432 and ±0.9408, with a turn number ratio of 4/9. According to the literature, the central region over which the magnetic-field uniformity reaches the 0.01% level is smaller than that of the Helmholtz configuration (0.268 vs. 0.38), whereas the regions corresponding to the 0.1% and 1% uniformity levels are both larger than those of the Helmholtz configuration. The magnetic-field series expansion, normalized to $B_{z}\left(0,0\right)$ and retained up to the second order, is

(13) $$B_{z}=1.000-2.410\times 10^{-3}z^{2}+1.205\times 10^{-3}\left(x^{2}+y^{2}\right).$$

Its second-order coefficient is about one order of magnitude larger than that of the Merritt configuration discussed below, indicating that its transverse-gradient contribution is less favorable for our application.

The Garrett coil (Figure 2b) consists of two pairs of circular coils [19,20,21]. The larger coils have a radius of 1, while the smaller coils have a radius of 0.6719. The larger coils are placed at z=±0.2976, and the smaller coils are placed at z=±0.7982. The turn number ratio between the larger and smaller coils is 22/15. The corresponding magnetic-field expansion is

(14) $$B_{z}=1.000-2.594\times 10^{-4}z^{2}+1.297\times 10^{-4}\left(x^{2}+y^{2}\right).$$

Among the configurations considered here, the Garrett configuration gives the smallest leading-order relative gradient. However, the need for coils with different radii makes the mechanical design and alignment more demanding.

The Merritt configuration [15] (Figure 2c) consists of four equal-sized square coils with a half-side length of 1. The four coils are placed at z=−0.5055, −0.1281, 0.1281, 0.5055, with a turn number ratio of 26:11:11:26. For the Merritt configuration used here, the calculated leading-order normalized field near the center is

(15) $$B_{x}=-4.174\times 10^{-4}xz,$$

(16) $$B_{y}=-4.174\times 10^{-4}yz,$$

(17) $$B_{z}=1-2.087\times 10^{-4}\left(x^{2}+y^{2}\right)+4.174\times 10^{-4}z^{2},$$

where *x*, *y*, and *z* are dimensionless coordinates normalized to the half-side length of the square coils. Although the Merritt configuration retains a second-order term, its coefficient is very small. Consequently, the relative gradient in the central region of interest is at the $10^{-5}$ level, which is sufficient for the polarized ^3^He cell used in this work.

Therefore, while the Garrett configuration provides the best field uniformity in terms of the leading-order gradient, the Merritt configuration provides a more practical solution because it uses equal-sized square coils and is much easier to fabricate, align, and integrate into the experimental apparatus. The expressions in Equations (15)–(17) are used as the basis for the field-uniformity estimate and for comparison with the experimental field maps.

The configuration selected here should therefore be understood as a solution optimized for the combined requirements of the present polarized ^3^He apparatus rather than as a claim of globally optimal field uniformity. Inverse-design methods may produce a larger homogeneous region, but the present system must simultaneously minimize the relative gradients relevant to ^3^He relaxation and satisfy practical constraints associated with optical access, the cell and oven, RF manipulation, magnetic shielding, heat load, fabrication, and alignment. The Merritt configuration provides a favorable balance of these requirements and enables the proposed second-order, single-axis characterization method to be experimentally tested in a simple and reproducible geometry.

The present analysis also suggests a physically motivated objective for future inverse design. Rather than minimizing only the conventional field variation, $\Delta B/B_{0}$, over a prescribed volume, the optimization can directly minimize the near-center expansion coefficients that determine the transverse gradients entering Equation (2). For specified ^3^He cell and operating conditions, these gradient terms can be weighted by the corresponding diffusion correlation functions (spectral densities), so that the objective function is directly related to the predicted gradient-induced relaxation rate. Target field and related inverse design methods could therefore optimize the coil geometry to achieve the resulting ^3^He relaxation time and could account for distortions induced by magnetic shields and surrounding structures. Developing such an application-specific inverse design procedure is beyond the scope of the present work and will be pursued in future studies.

## 3. Merritt Coil Design and Field Mapping Method

### 3.1. Coil Assembly

**Figure 3:**
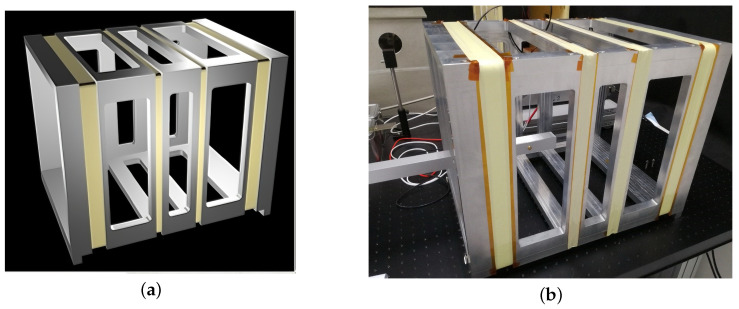
Merritt coil system. (**a**) Design rendering of coil system. (**b**) Photograph of fabricated system, with fluxgate magnetometer positioned at center to map magnetic field. Overall dimensions of system are approximately 41 cm × 41 cm × 53 cm.

The coil assembly was designed as a four-square-coil Merritt system (Figure 3a,b) to generate a holding field for polarized ^3^He experiments. The square geometry was selected because it provides good optical and mechanical access while maintaining high field uniformity in the central volume. The coil frame was fabricated from nonmagnetic aluminum alloy. The four square coils were mounted symmetrically about the central plane, and their turns were chosen according to the Merritt coil ratio. In the present implementation, the inner and outer coils were connected in series and driven by a low-noise DC current source.

In our earlier polarized ^3^He experiments, the holding field was typically generated using Helmholtz coils with diameters approaching 1 m. Although such coils provided the required field quality, their size and mass made relocation and reconfiguration difficult. The present four-square-coil system has a characteristic side length of approximately 40 cm and satisfies the required relative-gradient performance within the ^3^He cell volume while substantially reducing the overall dimensions of the apparatus. The complete assembly is sufficiently compact and lightweight to be moved by one person. This compactness and portability facilitate integration with optical pumping beams, the cell oven, RF coils, and magnetic shielding and constitute important practical advantages of the present implementation.

### 3.2. Magnetic-Field Mapping

The magnetic field was measured using a three-axis fluxgate magnetometer mounted on a motorized translation stage. The probe could be moved along the *x*, *y*, and *z* directions to obtain line profiles of the three field components. The representative parameters and the field-mapping setup are summarized in Table 2. As shown by the theoretical analysis, the coefficient governing the dominant second-order field gradients within the small central region can be determined from the variation in $B_{z}$ along the central symmetry axis. We therefore focus on the axial field profile in the following analysis. This reduced characterization does not imply that the complete higher-order three-dimensional field can be reconstructed from the axial scan alone.

Before establishing the near-center coefficient relations, we routinely characterized similar coil systems by mapping a three-dimensional region of approximately 30 cm × 30 cm × 30 cm. With a 1 cm step size, such a scan contains approximately 27,000 points and may require several days to complete; a modification of the coil parameters would require a comparable remapping. Under the symmetry and second-order near-center approximation derived above, the leading field-gradient coefficients relevant to the present ^3^He cell can instead be determined from an axial scan containing only approximately 30 points. This reduction applies to the dominant second-order gradients within the small central volume. Transverse and planar scans remain useful for diagnosing coil construction imperfections, probe misalignment, residual background fields, and higher-order non-axisymmetric contributions, but they are not required for extracting the leading coefficient used in the present relaxation assessment.

Accordingly, separate *x*- and *y*-axis line scans are not required to extract the dominant second-order gradients considered in this work. The use of an axial scan instead of routine complete three-dimensional mapping is a deliberate consequence of the near-center analysis rather than a limitation of the measurement system.

Because the field generated by the coil is superposed with ambient magnetic fields and possible probe offsets, two measurement protocols were considered. First, the field was measured with the coil current switched on and off, and the difference was used to estimate the coil-generated contribution. Second, scans were performed at a fixed current to evaluate the total field environment experienced by the ^3^He cell. The latter is particularly relevant for practical operation, where residual environmental fields and current-source stability also contribute to the effective holding field.

The field-normalized magnetic-field gradient was quantified as

(18) $$\epsilon \left(z\right)=\frac{1}{B_{z}\left(0\right)}\frac{\partial B_{z}}{\partial z},$$

where $B_{z}\left(0\right)$ is the central holding field.

## 4. Results

### 4.1. Axial Field Profile

**Figure 4:**
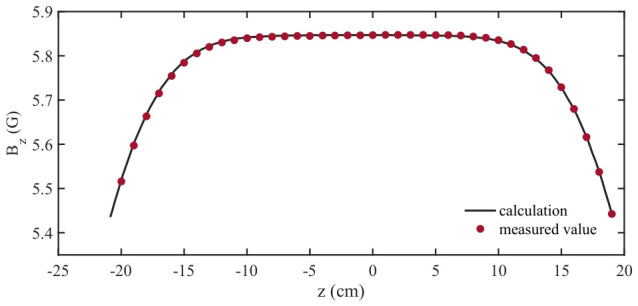
The solid dots show the measured $B_{z}$ field along the central axis with a step size of 1 cm, in units of gauss. The solid line represents the numerical calculation, fitted to the measurements with the current as the only adjustable parameter.

Figure 4 shows a representative axial scan of the magnetic field generated by the Merritt coil system. The measured result is compared with numerical calculations that account for the finite size of the winding wire. In the actual coil assembly, the four coils were wound with wire with a nominal diameter of 1.25 mm in 10 layers, with a turn count ratio of 26:11:11:26. Therefore, each coil consisted of multiple square loops with slightly different sizes and positions. For example, the outermost coil contained 260 turns and was modeled as 260 square loops. In the numerical calculation, the contributions from each loop were summed to obtain the final magnetic-field profile. This calculation is a direct numerical evaluation of the analytical vector potential solution rather than a finite-element simulation. The calculated result was fitted to the measured profile using the coil current as the only free parameter. The measured field profile is in good agreement with the calculated result over the full scan range. In the central region relevant to the ^3^He cell, the field remains highly uniform, as shown in the figure.

### 4.2. Measured Normalized Magnetic-Field Gradient

As shown in Equation (12), the measured axial variation in $B_{z}$ determines the coefficient *c* governing the dominant second-order near-center field components. The axial profile therefore provides a direct basis for evaluating the leading magnetic-field gradients within the central volume occupied by the ^3^He cell. It does not, however, determine the complete higher-order three-dimensional field of the square-coil system.

**Figure 5:**
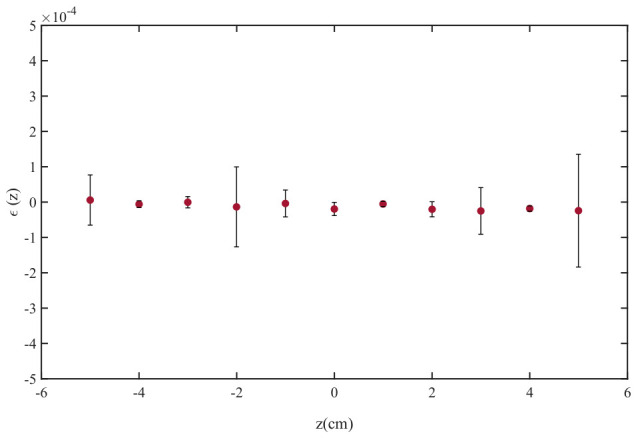
The measured normalized axial magnetic-field gradient in the central 10 cm interval of the Merritt coil system. This interval covers the approximately 5 cm polarized ^3^He cell used in the present apparatus.

The interval z∈[−5,5] cm was selected because the polarized ^3^He cell used in the present apparatus had a characteristic dimension of approximately 5 cm. The displayed 10 cm central interval therefore covers the complete cell volume with a substantial margin and should not be interpreted as the maximum useful uniform-field region of the coil system. Within this interval, the measured normalized magnetic-field gradients remain in the range of $10^{-5}$–$10^{-4}\;{\mathrm{cm}}^{-1}$, as shown in Figure 5. Considering the complete experimental uncertainty, we conservatively report the demonstrated performance at the $10^{-4}$ cm^−1^ level. This value is below the typical requirement of $3\times 10^{-4}$ cm^−1^ for polarized ^3^He experiments [1,22].

The measurements were performed at a holding field of approximately 5 G. The nominal precision of the three-axis fluxgate magnetometer was 10 nT, corresponding to a relative field scale of approximately $2\times 10^{-5}$ at this field strength. The practical field mapping accuracy was additionally limited by current-source fluctuations, magnetometer drift, cross-axis sensitivity, probe misalignment, positioning uncertainty, and time-dependent environmental magnetic fields. The laboratory is located approximately 100 m from a subway line and close to the University Motor Pool. The magnetometer was sufficiently sensitive to detect the magnetic disturbance produced by an individual truck passing nearby. Because different spatial positions were measured sequentially, such temporal background variations could appear as additional spatial scatter in the mapped field. The error bars therefore reflect the combined effects of the instrument, the measurement procedure, and the laboratory environment rather than the intrinsic field variation in the coil alone.

For comparison, in our earlier characterization of Helmholtz coil systems using a Hall probe, the limited resolution, temperature drift, and long-term instability of the probe allowed us to establish only that the normalized magnetic-field gradient was below approximately $3\times 10^{-4}$ cm^−1^ [22]. The present fluxgate measurements establish a gradient below $10^{-4}$ cm^−1^, and, therefore, they represent a substantial improvement in experimental characterization. The calculated field performance and practical constraints of the Helmholtz, Lee–Whiting, Garrett, and Merritt configurations are compared quantitatively in Table 1.

## 5. Discussion

We developed and experimentally characterized a four-square-coil Merritt system to produce a highly uniform holding field for polarized ^3^He experiments. Analytical modeling based on square-loop vector potentials and near-center symmetry expansion gives the leading-order field components relevant to gradient-induced ^3^He relaxation. The predicted near-center-field behavior was validated by three-axis fluxgate measurements, including axial scans and complete three-dimensional magnetic-field mapping.

The experimental results validate the usefulness of the Merritt four-square-coil configuration for polarized ^3^He experiments. Although a Garrett-type circular-coil system can provide a smaller calculated transverse gradient, it requires coils with different radii and nontrivial turn ratios. In contrast, the Merritt system uses square coils that are easier to fabricate, mount, and align, while still providing sufficient uniformity for the target ^3^He cell volume. The Merritt configuration should therefore be regarded as an application-specific compromise between field performance, optical and mechanical access, fabrication complexity, and compatibility with the surrounding experimental components.

The measured field profiles agree with the calculated field to within the level expected from the experimental uncertainties. The largest deviations appear in the off-center and edge regions of the mapped volume, where the field is more sensitive to probe positioning and background field subtraction. The central region, which is the relevant region for the ^3^He cell, shows much better agreement. In this region, the measured field nonuniformity reaches the $10^{-4}$ level, which is sufficient to suppress gradient-induced longitudinal relaxation.

An important methodological result of the present work is that the dominant second-order transverse gradients within the small central cell volume can be obtained from the axial curvature of $B_{z}$. Complete three-dimensional mapping remains useful for identifying construction imperfections, probe misalignment, environmental disturbances, and higher-order contributions, but it is not required for extracting the leading coefficient relevant to the present ^3^He relaxation analysis. This substantially reduces the time required for routine coil characterization and optimization.

The near-center expansion also provides a natural connection to target field and inverse design methods. Instead of minimizing only the conventional field variation, $\Delta B/B_{0}$, over a prescribed volume, a future optimization procedure can directly minimize the expansion coefficients that determine the transverse gradients and the corresponding ^3^He relaxation rate. The resulting optimized geometry can then be evaluated experimentally using the axial scan method developed here. Combining target-oriented coil design with the present near-center characterization framework may therefore lead to holding-field systems with even lower relaxation-inducing gradients while retaining the optical, mechanical, and shielding constraints of the apparatus.

The present measurement accuracy was also limited by time-dependent environmental magnetic noise. Because the spatial points were recorded sequentially, temporal disturbances from the nearby subway line and vehicle activity could appear as apparent spatial variations. To reduce this effect, we plan to develop an automated acquisition program that performs field scans during magnetically quiet night-time periods while simultaneously recording the coil current and ambient magnetic field. A stationary reference magnetometer and current-reversal measurements may further improve the subtraction of temporal background fields and instrumental offsets.

A Garrett coil system is currently being fabricated in our laboratory. This will allow a direct experimental comparison between the Merritt and Garrett configurations under the same measurement conditions. In addition to comparing their normalized magnetic-field gradients, we plan to place identical polarized ^3^He cells in the two systems and measure the resulting changes in $T_{1}$ and $T_{2}$. Such measurements will provide a more direct test of whether the smaller calculated leading-order gradient of the Garrett configuration produces a measurable improvement in the actual ^3^He relaxation performance.

In the longer term, higher-precision measurements may be performed inside a magnetically shielded room, where subway- and vehicle-related disturbances are strongly suppressed. Together with improved probe calibration, thermal stabilization, current monitoring, and automated night-time acquisition, this environment should permit a more stringent test of the calculated low-$10^{-5}$ gradient scale and of the differences among alternative coil configurations.

## 6. Conclusions

A compact four-square-coil Merritt system was designed, constructed, and experimentally characterized for polarized ^3^He precision measurements. The system provides the required normalized magnetic-field-gradient performance within the relevant cell volume, has a simple construction, and allows large optical and mechanical access. The near-center analysis further shows that the dominant second-order gradients can be determined from an axial field scan, thereby avoiding the routine need for complete three-dimensional mapping.

The main contribution of this work is therefore not the development of a new inverse design algorithm but an analysis and characterization method based on a near-center series expansion retained to the second order. By combining Maxwell’s equations with the spatial symmetry of the coil system, we show that, to this order, the near-center fields of both square- and circular-coil systems are determined by the axial variation in the field along the central symmetry axis. Consequently, a single-axis measurement of the axial field provides the information required to evaluate the dominant field-gradient effects relevant to polarized ^3^He, without requiring a complete three-dimensional field map of the region of interest. To the best of our knowledge, this framework has not previously been explicitly formulated for this purpose. To validate the analysis, we designed, constructed, and experimentally characterized a compact, mechanically accessible, and reproducible four-square-coil Merritt holding-field system tailored for polarized ^3^He precision measurements.

For ^3^He precision sensors, the main practical advantage of the Merritt coil system is not only its calculated uniformity but also its compatibility with optical access, ovens, laser beams, RF coils, and magnetic shielding. The design therefore provides a useful and reproducible platform for polarized noble gas magnetometers, neutron spin filters, comagnetometers, and other precision measurement experiments requiring a compact and uniform holding-field system.

## Figures and Tables

**Table 1:** Comparison of candidate coil configurations.

Configuration	Geometry	Leading Term and Estimated Gradient	Practical Consideration
Helmholtz	Two equal-radius circular coils	$\begin{array}{c} -1.152{\bar{z}}^{4}\\ 4.6\times 10^{-3} \end{array}$	Simple construction but relatively large fourth-order gradients away from center.
Lee–Whiting	Four equal-radius circular coils with unequal axial spacing and turn numbers	$\begin{array}{c} -2.410\times 10^{-3}{\bar{z}}^{2}\\ 4.8\times 10^{-4} \end{array}$	Equal coil radii but a larger leading gradient than the Garrett and Merritt configurations.
Garrett	Four circular coils with two different radii and a nontrivial turn ratio	$\begin{array}{c} -2.594\times 10^{-4}{\bar{z}}^{2}\\ 5.2\times 10^{-5} \end{array}$	Smallest leading-order gradient but more demanding fabrication and alignment.
Merritt	Four equal-sized square coils with a turn ratio of 26:11:11:26	$\begin{array}{c} +4.174\times 10^{-4}{\bar{z}}^{2}\\ 8.3\times 10^{-5} \end{array}$	Large optical access and simpler fabrication, mounting, and alignment.

The second line in the third column gives $\left|\partial \left(B_{z}/B_{0}\right)/\partial \bar{z}\right|$ evaluated at $|\bar{z}|=0.1$.

**Table 2:** Representative parameters of Merritt coil system and field mapping setup.

Parameter	Value
Coil type	Four-square-coil Merritt configuration
Measured coil constant	$k_{\exp}=9.164\pm 0.012$ G/A
Square-coil side length	∼40 cm
Frame material	Nonmagnetic aluminum alloy
Nominal holding field	∼1–50 G
Current source	Low-noise DC source
Magnetometer	Three-axis fluxgate magnetometer
	with nominal precision of 10 nT
Translation stage	Motorized three-axis translation stage with position precision
	of ∼10 μm and travel range of 50 cm for each axis
Mapping step size	1 cm for line scans; grid scan for two-dimensional maps
Central region of interest	Approximately [−5, 5] cm along each relevant coordinate

## Data Availability

The raw data supporting the conclusions of this article will be made available by the authors on request.
